# Response to Comment on Trends and outcomes of hospitalized patients with priapism in Germany: results from the GRAND study

**DOI:** 10.1038/s41443-024-00986-4

**Published:** 2024-10-01

**Authors:** Nikolaos Pyrgidis, Gerald B. Schulz, Julian Marcon

**Affiliations:** Department of Urology, University Hospital, LMU Munich, München, Germany

**Keywords:** Molecular biology, Medical research

We wish to thank Lindenbaum et al. for their valuable comment [1]. Indeed, priapism is a rare medical condition and large-scale studies on its prevalence and management are scarce [2]. Our study examines the patterns and clinical outcomes associated with priapism by providing crucial insights into the management of priapism based on data from the German Nationwide Inpatient Data (GRAND) [3].

The study demonstrated a steady increase in hospitalizations for low-flow priapism, while hospitalizations for high-flow priapism have decreased. Notably, low-flow priapism often necessitated surgical intervention, with 22.4% of cases requiring shunt surgery. Despite the guideline recommendations for early penile prosthesis implantation in cases of delayed or refractory low-flow priapism, only 2.5% of these patients received a penile prosthesis during their acute hospital stay. On the other hand, high-flow priapism was largely managed conservatively, with selective arterial embolization being performed in only 18.7% of cases, despite its favorable in-hospital outcomes. The latter is in line with the existing literature that endorses selective arterial embolization as a safe and effective treatment option in patients with high-flow priapism [4].

One of the critical observations in the study is the low rate of penile prosthesis implantation in patients undergoing shunt surgery for low-flow priapism. Current clinical guidelines advocate for early prosthesis implantation to prevent long-term erectile dysfunction and penile fibrosis [5]. The reluctance to perform this procedure in Germany could be attributed to various factors, including clinical conservatism or concerns about the complexity and risks of the procedure [6]. Accordingly, the high rate of exchange transfusions in patients with sickle cell disease-related priapism is another area where practice seems to diverge from guideline recommendations [7]. The observation that 37% of all patients with sickle cell disease received exchange transfusions suggests a need for a more tailored approach in this patient population.

The increase in low-flow priapism hospitalizations, contrasted with the decrease in high-flow cases, raises questions about the underlying causes. The decline in high-flow priapism cases may be attributed to better early intervention strategies and better management of pelvic injuries. Meanwhile, the rising incidence of low-flow priapism might reflect an increase in underlying conditions such as an exposure to medications or drugs, which are known risk factors for low-flow priapism [8]. Nevertheless, it should be highlighted that determining the underlying causes of the changing trends in priapism was beyond the scope of our analysis.

The findings from the GRAND study provide a robust basis for advocating changes in clinical practice in Germany and potentially other countries with similar healthcare systems. However, the study also highlights significant literature gaps that need to be addressed in future research. As priapism continues to be a challenging urological emergency with significant implications for patient outcomes, future research should focus on long-term outcomes and on the development of more personalized treatment approaches that align with the current guideline recommendations. As Lindenbaum et al. suggest, further high-volume studies at a global scale are mandatory. However, it should be noted that priapism is rare and studies exploring the trends and outcomes for this condition are difficult to conduct [9, 10]. Overall, due to the paucity of available studies, evidence deriving from administrative data may serve as a guide to improve clinical decision-making and patients’ outcomes, as well as to prompt healthcare providers to re-examine current practices in the management of priapism.
